# Multilevel Effects of Environmental and Neighborhood Factors on Sober Living House Resident 12-Month Outcomes

**DOI:** 10.15288/jsad.22-00307

**Published:** 2023-11-08

**Authors:** Meenakshi S. Subbaraman, Elizabeth Mahoney, Jane Witbrodt, Katherine J. Karriker-Jaffe, Amy A. Mericle, Douglas L. Polcin

**Affiliations:** ^a^ Behavioral Health and Recovery Studies, Public Health Institute, Oakland, California; ^b^ Alcohol Research Group, Emeryville, California; ^c^ Center on Behavioral Health Epidemiology, Implementation & Evaluation Research, RTI International, Berkeley, California

## Abstract

**Objective:**

Sober living houses (SLHs) are abstinence-based environments designed for individuals in recovery to live with others in recovery. Research shows that SLHs help some individuals maintain recovery and that certain SLH-related factors may be particularly protective. Here we assess how SLH housing and neighborhood characteristics are related to abstinence and psychiatric symptoms over time.

**Method:**

Baseline, 6-month, and 12-month data were collected from 557 SLH residents. Multilevel mixed models tested associations between house and neighborhood characteristics and individual-level percent days abstinent (PDA) and the number of psychiatric symptoms (measured with the Psychiatric Diagnostic Screening Questionnaire [PDSQ]) as outcomes. Final models adjusted for sex, age, and race/ ethnicity; ratings of house characteristics; and objective measurements of neighborhood-level exposures.

**Results:**

Both PDA and PDSQ improved significantly (*p*s ≤ .05) over time in both unadjusted and adjusted models. More self-help groups and fewer alcohol outlets within one mile were significantly protective for PDA, whereas walkability was significantly related to worse PDA and PDSQ (*p*s ≤ .05). For house-level factors, better ratings of house maintenance were related to significantly fewer psychiatric symptoms, whereas higher scores on SLH's safety measures and personal or residence identity were related to more psychiatric symptoms (*p*s ≤ .05). No house-level factor was significantly related to PDA.

**Conclusions:**

Neighborhood-level factors such as increased availability of self-help groups and fewer nearby alcohol outlets may increase abstinence among individuals living in SLHs. House-level factors related to better maintenance may also facilitate improved mental health.

Sober living houses (SLHs) are abstinence-based group residences designed for individuals who are attempting to maintain sobriety (Wittman & Polcin, 2014). SLHs do not provide professional services, such as counseling or treatment, but rather use a social model approach to recovery that emphasizes peer support and involvement in the household (Borkman, 1998; Polcin & Borkman, 2008). Research shows that SLHs help some individuals facilitate or maintain recovery; for example, residents maintain significant improvements in reduced substance use and psychiatric symptoms as well as other in other areas such as employment (Mericle et al., 2019; Polcin et al., 2010a, 2010b, 2018). For example, a study of 18-month outcomes found less substance use and fewer related problems in SLH residents over time (Polcin et al., 2010a), whereas another showed that psychiatric severity improved over the same period, and that psychiatric trajectories were related to abstinence (Polcin et al., 2016). Importantly, specific protective factors related to SLH house characteristics and the surrounding neighborhoods of SLHs remain understudied; thus, this study focuses on SLH house and neighborhood factors.

## Importance of house characteristics

For optimal functioning, the physical and social environment of a SLH should support social model recovery principles (Wittman et al., 2014; Polcin, Mahoney, & Mericle, 2021). Some have even framed the recovery process in SLHs as “the setting is the service” (Wittman et al., 2014). However, few studies have examined how specific house characteristics predict recovery outcomes. One study of SLH organizational characteristics found that residents who lived in SLHs that were part of a larger group of houses under one organization had increased odds of alcohol and drug abstinence (Mericle et al., 2019). Another recent study used the Recovery Home Environment Scale (RHES; Polcin, Mahoney, & Mericle, 2021), which assesses social model recovery characteristics of SLHs, to examine how SLH environmental characteristics related to the length of stay in the SLH (Mahoney et al., 2021). Results showed that sociability as measured by the RHES (e.g., resident support, empowerment, and involvement in house operations) was related to a longer length of stay, increased recovery capital (i.e., assets that aid recovery from substance use disorder), and reduced substance use.

In addition to providing a supportive environment, SLH architecture ideally encourages daily sober living and community-building (Wittman et al., 2014). However, studies of SLH physical and architectural characteristics are limited to descriptive and anecdotal reports. An SLH case report (Witt-man et al., 2014) noted key architectural characteristics such as building appearance and maintenance of the property; quality of furnishings; layouts that facilitate socializing with other residents, including outdoors; places to safely store personal possessions and similar security measures; and house policies that allow for expression of personal identity. This work influenced the development of the Recovery Home Architecture Scale (RHAS), a 25-item measure comprising six subscales that assess SLH architecture and how it supports peer-based recovery (Polcin et al., 2022). Yet while some evidence suggests that environmental and architectural characteristics of SLHs affect resident outcomes, no study has assessed how these factors affect long-term substance use or other recovery outcomes.

## Importance of neighborhood characteristics

The relationship between neighborhood factors and substance use outcomes in problem drinkers recruited from treatment was examined in a study testing a socioecological model of relapse and recovery, which found that neighborhood poverty and greater density of bars were related to a greater likelihood of relapse (Karriker-Jaffe et al., 2020). Outlet density is certainly related to alcohol consumption in the general population (Subbaraman et al., 2020), and restricting outlet density may be particularly relevant for those in recovery or who are attempting to reduce heavy drinking and its related consequences (Kerr & Subbaraman, 2022). Similarly, the density or availability of treatment facilities and self-help groups (e.g., Alcoholics Anonymous [AA]) in local neighborhoods might also influence recovery outcomes among SLH residents; for example, analyses of 6-month outcomes among SLH residents using the same sample as the current study found that availability of both AA and other self-help groups was related to increased abstinence (Mahoney et al., 2023).

A study describing neighborhood correlates of SLH locations found that several factors reflecting neighborhood socioeconomic disadvantage (e.g., rates of poverty and employment, median home values) were associated with whether neighborhoods had affordable SLHs, and that the strongest factor associated with affordable SLHs was the number of neighborhood treatment facilities (Mericle et al., 2016). The authors speculated that having SLHs close to treatment programs could benefit SLH residents, for example, through referrals to treatment programs that offer additional support to those who need it (Mericle et al., 2016).

A similar study describing the neighborhoods in which SLHs are located also found factors significantly associated with house location; for example, SLHs with higher fees were in neighborhoods with higher property values, fewer off-premise alcohol outlets, and greater distance from self-help groups than SLHs with lower fees (Mericle et al., 2020). The study also found that SLHs with larger capacity were in neighborhoods with more off-premise alcohol outlets but also better walkability and were closer to treatment facilities than SLHs with smaller capacity (Mericle et al., 2020). Given that SLHs were generally located in neighborhoods with both benefits and risk factors for recovery, the authors recommended that future work examine how similar neighborhood factors affect SLH resident outcomes (Mericle et al., 2016, 2020).

## Study aims and hypotheses

Research that has examined SLH characteristics (Mericle et al., 2019) and studies that have looked at facets of the house environment more specifically (Polcin, Mahoney, & Mericle, 2021; Polcin, Mahoney, Witbrodt, & Mericle, 2021) suggest that these are key factors in improving resident substance use outcomes. Studies that have examined neighborhood-level factors show that infrastructure has an impact on health outcomes and that SLHs are located in neighborhoods with both helpful and harmful characteristics. However, the relationships between these neighborhood characteristics and recovery outcomes among SLH residents have yet to be examined. Thus, our study aims were to assess how both SLH house and neighborhood characteristics are related to 12-month abstinence and psychiatric symptoms using longitudinal data from a sample of SLH residents. Based on existing evidence, we hypothesized that more positive ratings of house characteristics and higher scores on objective measurements, such as neighborhood walkability and self-help group availability, would be related to increased abstinence and fewer psychiatric symptoms. We also hypothesized that increased alcohol outlet density would be related to worse outcomes.

## Method

### Sample

We recruited 557 participants in 48 SLHs located in 44 neighborhoods in Los Angeles County, California. SLH selection purposively maximized the diversity of the socioeconomic status (SES) of the neighborhood in which the study SLHs are located. We approached an equal distribution of SLHs across SES quartiles, excluding houses that included residents’ children, houses that had fewer than six beds, houses that had more than 25 beds, and houses that had advertised fees that were more than $4,500 per month per person. Participants were required to be 18 years of age or older, provide informed consent, have a history of drug and/or alcohol problems, provide tracking information for follow-up interviews, and have been in the SLH for less than 1 month at baseline to ensure they were new residents. Study participants were enrolled from 2018 to 2021.

All new SLH residents (i.e., living in SLH for <30 days) were able to participate if they met inclusion/exclusion criteria. Study personnel worked with SLHs to determine optimal recruitment methods. Some preferred posting flyers, handing out brochures, or having the SLH manager pass along the interviewer's business card to the participant. Some SLHs requested in-person presentations at house meetings or to informally explain the study to new residents. We also offered $5 referral bonuses to participants who referred other residents to participate. If the new resident was interested, they could contact the house's assigned interviewer. Interviewers would also contact the house manager or owner on a regular basis to collect the number of new people who had entered the house. Throughout our recruitment period, approximately 987 new residents entered the 48 SLHs enrolled; 557 ultimately participated in this study. At study end, average length of stay in the baseline SLH was 169 days and median length of stay was 128 days.

The U.S. Census Bureau's American Community Surveys (ACS) were the primary source of data on neighborhood sociodemographic characteristics. These data are available for small areas such as Census tracts, which are the basic units we used to represent the neighborhood for each SLH. According to the ACS, the median gross rent for a one-bedroom apartment in LA County for 2016–2020 was $1,534 (census. gov). Of the 48 houses enrolled, 27.1% were from the low SES quartile, 20.8% from the second quartile, 27.1% from the third, and 25.0% from the highest. Twenty-four houses were for men, 11 for women, and 13 for all genders. Nine houses were affiliated with a treatment program and 26 were part of a larger organization of SLHs. All houses were members of the Sober Living Network (SLN), which provides member residences with support, guidance, advocacy, and certification for meeting national quality standards.

### Measures

*Outcome variables*. Our first outcome was percent days abstinent (PDA) from alcohol and drugs, because a primary goal of SLHs is to eliminate or at least reduce substance use. We used the Timeline Followback (Sobell et al., 1996) to collect daily substance use data to assess PDA from alcohol and drugs over the prior 6 months at baseline, again 6 months after study entry, and 12 months after study entry. Our second outcome was the number of psychiatric symptoms measured using the Psychiatric Diagnostic Screening Test (PDSQ; Zimmerman et al., 1999), also assessed at baseline, 6 months, and 12 months. The PDSQ counts symptoms related to 13 clinical disorders, including those common among persons with substance use disorders (e.g., depression, anxiety, post-traumatic stress disorder, and psychotic disorders). We calculated an overall PDSQ score (115 items across the 13 disorders) and used the natural log of PDSQ scores in regression analyses because of skewness in the distributions.

*Exposure variables*. Table 1 describes house and neighborhood exposure variables. To allow time for residents to form impressions of the specific SLH, we collected resident ratings of house characteristics at least 1 month after residents entered the SLHs. These measures were collected separately from the baseline interview, at least 1 month but at most 3 months after house entry and were measured only once; we did not consider these impressions constant over time and thus interpreted results as how their scores at this time point affected later outcomes.

**Table 1.**
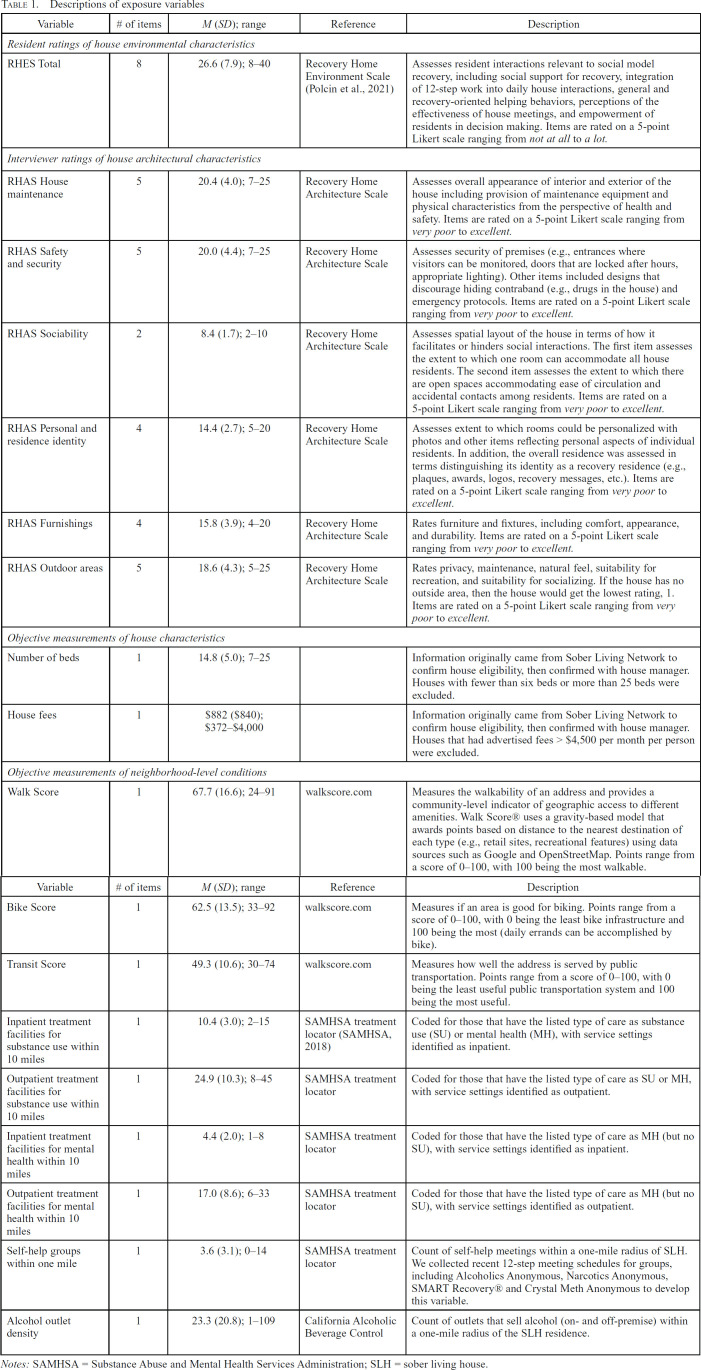
Descriptions of exposure variables

Variable	# of items	M (SD); range	Reference	Description
Resident ratings of house environmental characteristics				
RHES Total	8	26.6 (7.9); 8-40	Recovery Home Environment Scale (Polcin et al., 2021)	Assesses resident interactions relevant to social model recovery, including social support for recovery, integration of 12-step work into daily house interactions, general and recovery-oriented helping behaviors, perceptions of the effectiveness of house meetings, and empowerment of residents in decision making. Items are rated on a 5-point Likert scale ranging from *not at all to a lot*.
Interviewer ratings of house architectural characteristics				
RHAS House maintenance	5	20.4 (4.0); 7-25	Recovery Home Architecture Scale	Assesses overall appearance of interior and exterior of the house including provision of maintenance equipment and physical characteristics from the perspective of health and safety. Items are rated on a 5-point Likert scale ranging from *very poor to excellent*.
RHAS Safety and security	5	20.0 (4.4); 7-25	Recovery Home Architecture Scale	Assesses security of premises (e.g., entrances where visitors can be monitored, doors that are locked after hours, appropriate lighting). Other items included designs that discourage hiding contraband (e.g., drugs in the house) and emergency protocols. Items are rated on a 5-point Likert scale ranging from *very poor to excellent*.
RHAS Sociability	2	8.4 (1.7); 2-10	Recovery Home Architecture Scale	Assesses spatial layout of the house in terms of how it facilitates or hinders social interactions. The first item assesses the extent to which one room can accommodate all house residents. The second item assesses the extent to which there are open spaces accommodating ease of circulation and accidental contacts among residents. Items are rated on a 5-point Likert scale ranging from *very poor to excellent*.
RHAS Personal and residence identity	4	14.4 (2.7); 5-22	Recovery Home Architecture Scale	Assesses extent to which rooms could be personalized with photos and other items reflecting personal aspects of individual residents. In addition, the overall residence was assessed in terms distinguishing its identity as a recovery residence (e.g., plaques, awards, logos, recovery messages, etc.). Items are rated on a 5-point Likert scale ranging from *very poor to excellent*.
RHAS Furnishings	4	15.8 (3.9); 4-22	Recovery Home Architecture Scale	Rates furniture and fixtures, including comfort, appearance, and durability. Items are rated on a 5-point Likert scale ranging from *very poor to excellent*.
RHAS Outdoor areas	5	18.6 (4.3); 5-25	Recovery Home Architecture Scale	Rates privacy, maintenance, natural feel, suitability for recreation, and suitability for socializing. If the house has no outside area, then the house would get the lowest rating, 1. Items are rated on a 5-point Likert scale ranging from *very poor to excellent*.
Objective measurements of house characteristics				
Number of beds	1	14.8 (5.0); 7-25		Information originally came from Sober Living Network to confirm house eligibility, then confirmed with house manager. Houses with fewer than six beds or more than 25 beds were excluded.
House fees	1	$882 ($840);$372-$4,000		Information originally came from Sober Living Network to confirm house eligibility, then confirmed with house manager. Houses that had advertised fees > $4,500 per month per person were excluded.
Objective measurements of neighborhood-level conditions				
Walk Score	1	67.7(16.6); 24-91	walkscore.com	Measures the walkability of an address and provides a community-level indicator of geographic access to different amenities. Walk Score@ uses a gravity-based model that awards points based on distance to the nearest destination of each type (e.g., retail sites, recreational features) using data sources such as Google and OpenStreetMap. Points range from a score of 0-100, with 100 being the most walkable.
Bike Score	1	62.5 (13.5); 33-92	walkscore.com	Measures if an area is good for biking. Points range from a score of 0-100, with 0 being the least bike infrastructure and 100 being the most (daily errands can be accomplished by bike).
Transit Score	1	49.3 (10.6); 30-74	walkscore.com	Measures how well the address is served by public transportation. Points range from a score of 0-100, with 0 being the least useful public transportation system and 100 being the most useful.
Inpatient treatment facilities for substance use within				
10 miles	1	10.4 (3.0); 2-15	SAMHSA treatment locator (SAMHSA, 2018)	Coded for those that have the listed type of care as substance use (SU) or mental health (MH), with service settings identified as inpatient.
Outpatient treatment facilities for substance use within				
10 miles	1	24.9(10.3); 845	SAMHSA treatment locator	Coded for those that have the listed type of care as SU or MH, with service settings identified as outpatient.
Inpatient treatment facilities for mental health within 10 miles	1	4.4 (2.0); 1-8	SAMHSA treatment locator	Coded for those that have the listed type of care as MH (but no SU), with service settings identified as inpatient.
Outpatient treatment facilities for mental health within 10 miles	1	17.0 (8.6); 6-33	SAMHSA treatment locator	Coded for those that have the listed type of care as MH (but no SU), with service settings identified as outpatient.
Self-help groups within one mile	1	3.6 (3.1); 0-14	SAMHSA treatment locator	Count of self-help meetings within a one-mile radius of SLH. We collected recent 12-step meeting schedules for groups, including Alcoholics Anonymous, Narcotics Anonymous, SMART Recovery@ and Crystal Meth Anonymous to develop this variable.
Alcohol outlet density	1	23.3 (20.8); 1-109	California Alcoholic Beverage Control	Count of outlets that sell alcohol (on- and off-premise) within a one-mile radius of the SLH residence.

*Notes:* SAMHSA = Substance Abuse and Mental Health Services Administration; SLH = sober living house.

Trained interviewers rated the architectural features of the house using the Recovery House Architecture Scale (RHAS; Polcin et al., 2023). Table 1 describes the RHAS subscales as well as two objective measurements of house characteristics—number of beds and house fees. We also obtained objective measurements of neighborhood conditions from sources listed in Table 1, all using 2018 data. For treatment facilities and self-help group meetings, facility and meeting locations were geocoded and mapped in relation to each SLH in the sample to create the density measures (e.g., number of treatment facilities within 10 miles of an SLH). A similar process was used to map alcohol outlets in relation to each SLH.

*Statistical analyses*. We first examined resident, house, and neighborhood characteristics using descriptive statistics and used bivariate chi-square and *t* tests to assess differences in attrition by these characteristics. We then used longitudinal mixed-effect models to examine changes in PDA and PDSQ (separately) from baseline to 12 months. We tested the relationships between each exposure and outcome variable in separate longitudinal mixed-effect models, adjusted for time (interview), resident gender, age, and race/ethnicity, with random effects for neighborhood and person and using robust standard errors. The inclusion of the random effect for neighborhood adjusts for clustering within houses because of the minimal overlap of houses within neighborhoods (48 houses across 44 neighborhoods). An additional random effect for house could not be included because of this col-linearity; sensitivity analyses including a random effect for house instead of neighborhood showed no differences. Additional sensitivity analyses showed that results were robust to inclusion of resident's past-12-month income, which was ultimately left out of models for parsimony.

Exposure variables that were significant at *p* ≤ .05 when examined separately were retained for testing in the final combined models. All analyses were performed using Stata v17 (StataCorp LP, College Station, TX).

## Results

### Demographic characteristics and changes in outcomes over time

**Figure 1.**
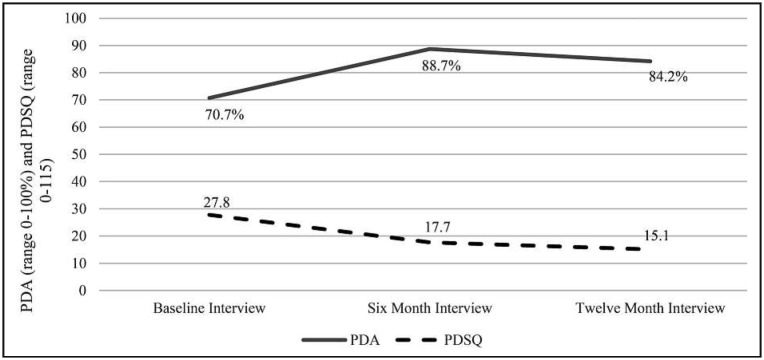
Unadjusted percent days abstinent (PDA) and number of Psychiatric Diagnostic Screening Test Score (PDSQ) symptoms over time, sample of *N* = 557 residents living in sober living houses, Los Angeles County 2018–2021

Table 2 describes baseline demographic characteristics of our sample of SLH residents by interview (baseline, 6 months, and 12 months) and indicates high follow-up rates (83% at 6 months and 82% at 12 months). Bivariate tests showed minimal bias attributable to attrition, with the only significant (*p* < .05) difference being that proportionately more women than men completed the 12-month interview versus not. Most importantly, there were no significant differences in baseline measures of outcomes for those who completed the 12-month interview (*n* = 456) versus those lost to follow-up (*n* = 101, data not shown). Figure 1 illustrates unadjusted changes in PDA and PDSQ over time, with the vertical axis representing both PDA (0%–100%) and PDSQ (i.e., psychiatric symptom count, 0–115). Ultimately, residents improved in both outcomes: average PDA for the past 6 months increased from 70.7% at baseline to 84.2% at 12 months, and average PDSQ decreased from 27.8 symptoms at baseline to 15.1 symptoms at 12 months.

**Table 2.**
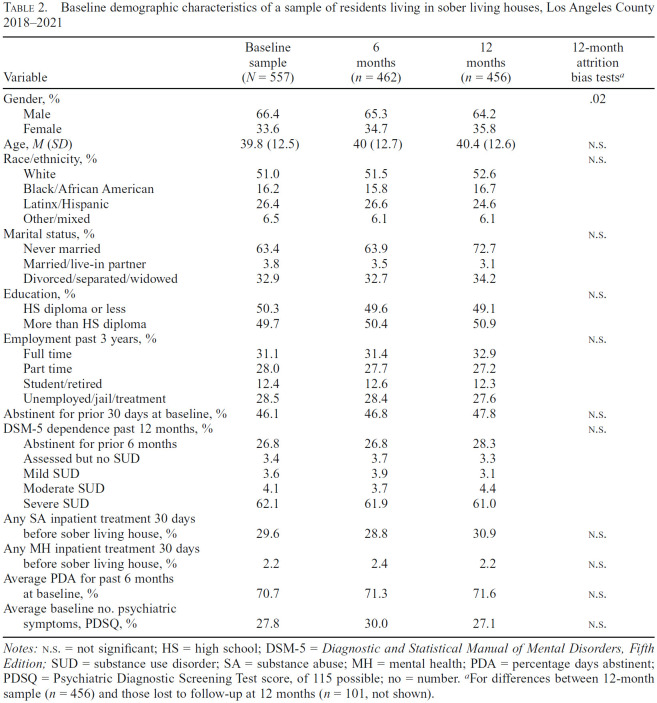
Baseline demographic characteristics of a sample of residents living in sober living houses, Los Angeles County 2018–2021

Variable	Baseline sample (N = 557)	6 months (n = 462)	12 months (n = 456)	12-months attrition bias tests^a^
Gender, %				.02
Male	66.4	65.3	64.2	
Female	33.6	34.7	35.8	
Age, M (SD)	39.8(12.5)	40 (12.7)	40.4 (12.6)	n.s.
Race/ethnicity, %				n.s.
White	51.0	51.5	52.6	
Black/African American	16.2	15.8	16.7	
Latinx/Hispanic	26.4	26.6	24.6	
Other/mixed	6.5	6.1	6.1	
Marital status, %				n.s.
Never married	63.4	63.9	72.7	
Married/live-in partner	3.8	3.5	3.1	
Divorced/separated/widowed	32.9	32.7	34.2	
Education, %				n.s.
HS diploma or less	50.3	49.6	49.1	
More than HS diploma	49.7	50.4	50.9	
Employment past 3 years, %				n.s.
Full time	31.1	31.4	32.9	
Part time	28.0	27.7	27.2	
Student/retired	12.4	12.6	12.3	
Unemployed/jail/treatment	28.5	28.4	27.6	
Abstinent for prior 30 days at baseline, %	46.1	46.8	47.8	n.s.
DSM-5 dependence past 12 months, %				n.s.
Abstinent for prior 6 months	26.8	26.8	28.3	
Assessed but no SUD	3.4	3.7	3.3	
Mild SUD	3.6	3.9	3.1	
Moderate SUD	4.1	3.7	4.4	
Severe SUD	62.1	61.9	61.0	
Any SA inpatient treatment 30 days before sober living house, %	29.6	28.8	30.9	n.s.
Any MH inpatient treatment 30 days before sober living house, %	2.2	2.4	2.2	n.s.
Average PDA for past 6 months at baseline, %	70.7	71.3	71.6	n.s.
Average baseline no. psychiatric symptoms, PDSQ, %	27.8	30.0	27.1	n.s.

*Notes:* n.s. = not significant; HS = high school; DSM-5 = *Diagnostic and Statistical Manual of Mental Disorders, Fifth Edition;* SUD = substance use disorder; SA = substance abuse; MH = mental health; PDA = percentage days abstinent; PDSQ = Psychiatric Diagnostic Screening Test score, of 115 possible; no = number.

a For differences between 12-month sample (*n* = 456) and those lost to follow-up at 12 months (*n* = 101, not shown).

### Associations between SLH house and neighborhood characteristics and abstinence

Table 3 shows results from longitudinal mixed-effect models predicting PDA (Model 1) and PDSQ (Model 2) from neighborhood and house exposures, adjusted for resident sex, age, and race/ethnicity. These models simultaneously include exposure variables that were significant at *p* ≤ .05 when examined as individual exposure variables in separate regression models (Supplemental Table). (Supplemental material appears as an online-only addendum to this article on the journal's website.) Exposure variables that were not significant in the separate regression models are marked with a dash in Table 3; for example, the RHES was not significant in separate regression models (Supplemental Table) and thus not included in Table 3 models. We also checked the variance inflation factors (VIFs) for both models. Although none of the variables in the model for PDA had a VIF greater than 10, a few exposures did have VIFs greater than 10 in the model for PDSQ; these were removed from the final model accordingly and are marked with dashes in Table 3.

**Table 3.**
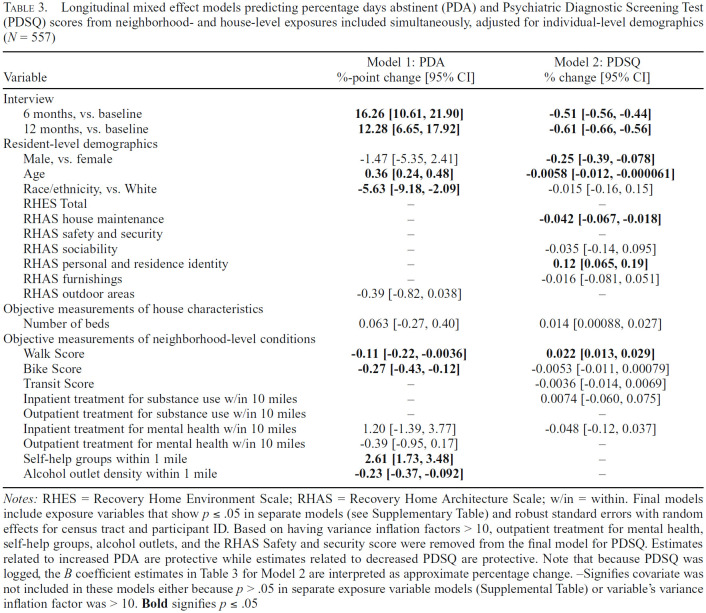
Longitudinal mixed effect models predicting percentage days abstinent (PDA) and Psychiatric Diagnostic Screening Test (PDSQ) scores from neighborhood- and house-level exposures included simultaneously, adjusted for individual-level demographics (*N* = 557)

Variable	Model 1: PDA %-point change [95% CI]	Model 2: PDSQ % change [95% CI]
Interview		
6 months, vs. baseline	**16.26 [10.61,21.90]**	**-0.51 [-0.56, -0.44]**
12 months, vs. baseline	**12.28 [6.65,17.92]**	**-0.61 [-0.66, -0.56]**
Resident-level demographics		
Male, vs. female	-1.47 [-5.35, 2.41]	**-0.25 [-0.39, -0.078]**
Age	**0.36 [0.24, 0.48]**	**-0.0058 [-0.012, -0.000061]**
Race/ethnicity, vs. White	**-5.63 [-9.18,-2.09]**	-0.015 [-0.16, 0.15]
RHES Total	-	-
RHAS house maintenance	-	**-0.042 [-0.067, -0.018]**
RHAS safety and security		
RHAS sociability	-	-0.035 [-0.14, 0.095]
RHAS personal and residence identity	.-	**0.12 [0.065, 0.19]**
RHAS furnishings		-0.016 [-0.081, 0.051]
RHAS outdoor areas	-0.39 [-0.82, 0.038]	
Objective measurements of house characteristics		
Number of beds	0.063 [-0.27, 0.40]	0.014 [0.00088, 0.027]
Objective measurements of neighborhood-level conditions		
Walk Score	**-0.11 [-0.22, -0.0036]**	**0.022 [0.013, 0.029]**
Bike Score	**-0.27 [-0.43, -0.12]**	-0.0053 [-0.011, 0.00079]
Transit Score	-	-0.0036 [-0.014, 0.0069]
Inpatient treatment for substance use w/in 10 miles	-	0.0074 [-0.060, 0.075]
Outpatient treatment for substance use w/in 10 miles	-	-
Inpatient treatment for mental health w/in 10 miles	1.20 [-1.39, 3.77]	-0.048 [-0.12, 0.037]
Outpatient treatment for mental health w/in 10 miles	-0.39 [-0.95, 0.17]	-
Self-help groups within 1 mile	**2.61 [1.73, 3.48]**	.-
Alcohol outlet density within 1 mile	**-0.23 [-0.37, -0.092]**	.-

*Notes*: RHES = Recovery Home Environment Scale; RHAS = Recovery Home Architecture Scale; w/in = within. Final models include exposure variables that show *p* ≤ .05 in separate models (see Supplementary Table) and robust standard errors with random effects for census tract and participant ID. Based on having variance inflation factors > 10, outpatient treatment for mental health, self-help groups, alcohol outlets, and the RHAS Safety and security score were removed from the final model for PDSQ. Estimates related to increased PDA are protective while estimates related to decreased PDSQ are protective. Note that because PDSQ was logged, the *B* coefficient estimates in Table 3 for Model 2 are interpreted as approximate percentage change.–Signifies covariate was not included in these models either because *p* > .05 in separate exposure variable models (Supplemental Table) or variable's variance inflation factor was > 10. **Bold** signifies *p* ≤ .05

As illustrated in Figure 1, Table 3 shows significant (*p* ≤ .05) improvements in PDA at both follow-ups relative to baseline, with a large improvement between baseline and 6 months (16.26 percentage point increase on average, 95% CI [10.61, 21.90]), and a slightly smaller improvement maintained between baseline and 12 months (12.28 percentage point increase on average, 95% CI [6.65, 17.92]). None of the resident or interviewer ratings of house characteristics were significantly related to PDA in separate models (Supplemental Table) except for the RHAS Outdoor areas measure, which did not remain significant in the final model for PDA (Table 3, Model 1). Number of beds in the SLH was no longer significant either. However, a number of objective measurements of neighborhood-level exposures from the separate regression models (Supplemental Table) did remain significantly associated with PDA in the final model. Higher (better) Walk and Bike Scores were associated with slightly lower PDA, with an average decrease of .11 and .27 percentage points in PDA for each 1-point increase in the neighborhood Walk and Bike Scores, respectively. Increased availability of self-help groups within 1 mile was associated with higher PDA, whereas more alcohol outlets within 1 mile were associated with lower PDA. Specifically, one additional self-help group within 1 mile of the SLH was related to a 2.61 average percentage point increase in PDA (95% CI [1.73, 3.48]) whereas one additional alcohol outlet within 1 mile was related to a .23 average percentage point decrease in PDA (95% CI [-.37, -.092]).

### Associations between SLH house and neighborhood characteristics and psychiatric symptoms

Results for PDSQ showed some similar and some distinct patterns compared with results for PDA (Table 3, Model 2). Note that because PDSQ was logged, the estimates shown in Table 3 for Model 2 were converted and are interpreted as percent change. Like PDA, PDSQ improved significantly over time, yet with smaller improvements between baseline and 6 months (51% average decrease in symptom count, 95% CI [-56%, -44%]), and slightly larger improvements between baseline and 12 months (61% average decrease in symptom count, 95% CI [-66%, -56%]). Unlike for PDA, interviewer ratings of house characteristics were significantly related to PDSQ in both the separate regression models (Supplemental Table) and final regression model: specifically, higher scores on house maintenance were related to lower (better) PDSQ whereas higher scores on personal and residence identity were related to higher (worse) PDSQ. For objective measurements of neighborhood-level exposures, only higher neighborhood Walk Scores remained related to slightly higher PDSQ (*p*s ≤ .05) in the final model, with a 22% average increase in PDSQ for each 1-point increase in the Walk Score.

## Discussion

We examined how SLH and neighborhood characteristics are related to abstinence and psychiatric symptoms using longitudinal data from a sample of SLH residents. Results show significant improvements in both abstinence and psychiatric symptoms over time, and that each outcome has some similar and some distinct relationships with house- and neighborhood-level exposures.

### House-level factors

We hypothesized that more positive ratings of house architectural characteristics would be related to increased abstinence and fewer psychiatric symptoms. Although none of the house-level characteristics were significantly related to PDA, two were related to PDSQ, perhaps reflecting that psychiatric symptoms might be more sensitive to house architecture than substance use. First, better scores on SLH maintenance were associated with fewer psychiatric symptoms, suggesting that the appearance and physical characteristics of SLHs might affect residents’ mental health. Given that measures of well-being beyond substance use are crucial indicators of recovery (Kaskutas et al., 2014), this finding is particularly important to consider when prioritizing the costs of maintaining SLHs. For example, SLHs might consider procedures for ensuring good maintenance by involving residents in basic activities such as cleaning. Perhaps part of better mental health is resident commitment, sense of accomplishment, and connection to the household when they help maintain it. Prior analyses have highlighted peer involvement in household activities as one of the SLH social environmental factors most strongly related to increased recovery capital (Polcin, Mahoney, Witbrodt, & Mericle, 2021), and future research should further investigate how involvement in SLH maintenance is related to recovery outcomes, perhaps through qualitative interviews with house residents.

Unexpectedly, having a stronger SLH personal and residence identity was related to more psychiatric symptoms. It is unclear why living in an SLH that encourages resident personalization would be associated with psychiatric symptoms, but perhaps these personal identity markers create conflict between residents because of different styles or beliefs. The unexpected result for the RHAS personal and residence identity indicator could also be attributable to an unexpected association with one of the individual items within the subscale (e.g., the item measuring identity as a recovery residence and not items measuring extent to which rooms could be personalized). For example, SLHs that distinguish themselves more than others (e.g., with physical plaques, logos) may be reminding residents that they are away from home, leading to distress. Future analyses will examine relationships between psychiatric symptoms and individual RHAS items measuring SLH personal and residence identity to understand what is driving these unexpected associations.

### Neighborhood-level factors

As hypothesized, greater alcohol outlet density was significantly related to less abstinence in our final model and to more psychiatric symptoms in preliminary models. Contrary to hypotheses, neighborhood walkability was also related to worse outcomes. The Walk Score measures pedestrian friendliness and indicates ease of accessing amenities. SLHs are often found in neighborhoods with both high walkability and high alcohol outlet density (Mericle et al., 2020), and the correlation between neighborhood Walk Score and alcohol outlet density in this sample was substantial (.62, analyses not shown); alcohol outlet density was ultimately dropped from the final model because of multicollinearity. Still, the significant relationship between walkability and worse outcomes might reflect easier access to alcohol and/or other substances. Studies on alcohol policy and drinking in the general population support that increased alcohol outlet density is related to worse alcohol-related outcomes (Subbaraman et al., 2020), and some have argued that reducing outlet density and banning advertising may be particularly important policies for individuals maintaining recovery (Kerr & Subbaraman, 2022).

Although results did not remain significant in final models adjusted for other exposures, preliminary models showed that more inpatient and outpatient treatment facilities for mental health within 10 miles of the SLH were related to both increased abstinence and fewer psychiatric symptoms. Local SLHs may have informal relationships or affiliations with nearby treatment programs, and geographic proximity might make it easier for residents to attend programs at those facilities. Because we do not have these data, future studies could examine how SLH affiliations with treatment facilities are associated with resident outcomes. Similarly, availability of self-help groups was significantly related to increased abstinence. This finding was expected, as self-help group attendance has been repeatedly linked to better outcomes (Donovan et al., 2013). However, because we did not examine residents’ self-help group attendance or level of involvement here, future studies are needed to confirm that the relationship between self-help group availability and positive outcomes is actually attributable to involvement in neighborhood self-help groups.

### Strengths and limitations

Primary strengths include the use of longitudinal data from a sample with a high follow-up rate (82% at 12 months). Analyses also used both objective measurements of neighborhood exposure variables and individual-level ratings, which alleviates concerns regarding measurement error. Still, results come with limitations. First, although the improvements in outcomes over time observed here mirror results from previous SLH studies (Jason et al., 2007; Mericle et al., 2019; Polcin et al., 2010, 2018; Tuten et al., 2012), our findings should not be viewed as indications of SLH effectiveness per se because we do not have a comparison group of individuals not living in SLHs. Second, we are unable to calculate sampling proportions or meaningfully assess the generalizability of our SLH sample. SLHs are not licensed or required to report their existence to any government agency, and it is therefore impossible to know the exact number of residences in a given area. We recruited SLHs that were members of SLH associations such as the SLN and California Consortium of Addiction Programs and Professionals, which report a combined membership of nearly 800 houses in the state (Wittman & Polcin, 2014). However, this study was conducted in one type of recovery housing in one region of California, where the SLHs were allowed to decide on participation and were all part of the SLN. This self-selection bias may mean that the houses that chose to participate may not represent the broad diversity of SLHs. Future studies could expand beyond those SLHs that are accredited by an organization, in different geographic areas, and to other types of recovery housing. Finally, although attrition was generally minimal and not related to outcomes, we did find differential attrition by gender and adjusted for gender in all regression models.

### Conclusions

Neighborhood-level factors such as increased availability of self-help groups and fewer alcohol outlets may be protective for individuals living in SLHs. House-level factors related to better maintenance and social support might also protect against psychiatric symptoms. Providers who are deciding on locations for new houses, expanding existing SLH sites, or modifying architectural designs of current houses should consider the neighborhood and architectural factors found to be associated with outcomes. Results regarding architectural and neighborhood factors can help SLH operators enhance or change the environments within the house or where it is located. This information can also help guide persons seeking a suitable SLH for their own residence.
